# Evaluation of facial pleasantness in patients with complete and unilateral cleft lip and palate rehabilitated and submitted to orofacial harmonization

**DOI:** 10.1590/2177-6709.29.1.e2423115.oar

**Published:** 2024-03-29

**Authors:** Daniel Giaretta FANGUEIRO, Daniela Gamba GARIB, Ana Cláudia de Castro Ferreira CONTI, Lucila LARGURA

**Affiliations:** 1 Universidade de São Paulo, Hospital de Reabilitação de Anomalias Craniofaciais (Bauru/SP, Brazil).; 2 Universidade do Sagrado Coração, Faculdade de Odontologia (Bauru/SP, Brazil).; 3 Universidade de São Paulo, Faculdade de Odontologia de Bauru (Bauru/SP, Brazil).; 4 Universidade Norte do Paraná, Faculdade de Odontologia (Londrina/PR, Brazil).; 5 Faculdade ILAPEO (Curitiba/PR, Brazil).

**Keywords:** Cleft lip and palate, Hyaluronic acid, Dermal filler, Facial pleasantness, Fissura labiopalatina, Ácido hialurônico, Preenchimento dérmico, Agradabilidade facial

## Abstract

**Objective:**

The objective of the present prospective case control study was to evaluate the facial pleasantness of patients with complete and unilateral cleft lip and palate at the end of interdisciplinary rehabilitation, submitted to facial fillers based on hyaluronic acid.

**Methods:**

The study group consisted of 18 individuals with complete unilateral cleft lip and palate, aged between 18 and 40 years (mean age 29 years) of both sexes. The patients presented a concave profile with mild to moderate maxillary deficiency, with completed orthodontic treatment and conducted by means of dentoalveolar compensations without orthognathic surgery. Participants underwent facial filling procedures with hyaluronic acid (HA) in the midface, inserted by a single operator. Standard photographs in frontal norm at rest, right profile at rest, and left profile at rest were obtained from each patient at the following operative times: (T1) pre-filler and (T2) and one-month post-filler. The photographs in T1 and T2 were randomly placed on a page of a virtual album. A 5-point Likert scale was used to assess facial pleasantness. The photographs were evaluated by two groups of evaluators consisting of 18 individuals with cleft lip and palate (CLPG=18) and 18 orthodontists with experience in the treatment of clefts (OG=18). For comparison between phases T1 and T2, and between evaluators with orofacial clefts and orthodontists, the Wilcoxon test was used (*p*<0,05).

**Results:**

People with cleft lip and palate rated their face as more pleasant after the midface filling procedure. In the perception of the orthodontists, on the other hand, the facial pleasantness remained similar after the facial filling procedure.

**Conclusions:**

The filling of the middle third of the face in patients with cleft lip and palate treated without orthognathic surgery increased the pleasantness of the face in the opinion of laypeople with cleft lip and palate.

## RESUMO

**Objetivo:**

O objetivo do presente estudo caso-controle prospectivo foi avaliar a agradabilidade facial de pacientes com fissura labiopalatina completa e unilateral ao final da reabilitação interdisciplinar, submetidos a preenchimentos faciais à base de ácido hialurônico.

**Métodos:**

O grupo de estudo foi composto por 18 indivíduos com fissura labiopalatina unilateral completa, com idade entre 18 e 40 anos (média de 29 anos), de ambos os sexos. Os pacientes apresentavam perfil côncavo com deficiência maxilar leve a moderada, com tratamento ortodôntico concluído e realizado por meio de compensações dentoalveolares sem cirurgia ortognática. Os participantes foram submetidos a procedimentos de preenchimento facial com ácido hialurônico (AH) no terço médio da face, implantado por um único operador. Fotografias padrão em norma frontal em repouso, perfil direito em repouso e perfil esquerdo em repouso foram obtidas de cada paciente nos seguintes tempos operatórios: (T1) pré-preenchimento e (T2) um mês pós-preenchimento. As fotografias em T1 e T2 foram inseridas aleatoriamente em uma página de um álbum virtual. Uma escala Likert de 5 pontos foi utilizada para avaliar a agradabilidade facial. As fotografias foram avaliadas por dois grupos de avaliadores formados por 18 indivíduos com fissura labiopalatina (GFLP=18) e 18 ortodontistas com experiência no tratamento de fissuras (GO=18). Para comparação entre as fases T1 e T2, e entre avaliadores com fissura labiopalatina e ortodontistas, foi utilizado o teste de Wilcoxon (*p*<0,05).

**Resultados:**

As pessoas com fissura labiopalatina avaliaram seu rosto como mais agradável após o preenchimento do terço médio da face. Já na percepção do ortodontista, a agradabilidade facial permaneceu semelhante após o procedimento de preenchimento facial.

**Conclusões:**

O preenchimento do terço médio da face em pacientes com fissura labiopalatina tratados sem cirurgia ortognática aumentou a agradabilidade da face na opinião dos leigos com fissura labiopalatina.

## INTRODUCTION

Cleft lip and palate (CLP) represent the most prevalent craniofacial malformations in humans.^1^ It may involve lips, alveolar ridge, and palate, with aesthetic, functional, and psychosocial consequences.^2^ Its origin is related to the embryonic period of intrauterine life, and it have a multifactorial aetiology, associating genetic and environmental factors.^3^ The rehabilitation of these patients is complex, and multidisciplinary, and begins on average at three months of life. The rehabilitation protocol for these patients includes lip, nose and palate repair, alveolar bone grafting, and orthodontics, isolated or combined with orthognathic surgery.^4^ ^,^ ^5^ ^,^ ^6^ The morphological sequelae of these primary surgeries (cheiloplasty and palatoplasty) influence craniofacial growth mainly by three-dimensional restriction of maxillary development. This maxillary underdevelopment contributes to the occurrence of Class III occlusal relationships, anterior crossbite and midface retrusion, and is related to speech-disorders and respiratory and facial disharmonies.^7^

Despite the obvious aesthetic and functional benefits at the end of this sequence of therapies, the expectations of facial aesthetic corrections for these patients are often not fully met. The most stigmatizing features that have been referred to as subject to correction at the end of surgical treatments are inadequate projection and contour of the upper lip, implantation of the nose, and maxillary atresia. In addition, the desire for less invasive procedures is increasing, considering the possibilities of corrections currently available and publicized.^8^

Hyaluronic acid has been widely used as facial filler. This product is indicated for specific corrections and restores a youthful appearance to the face, projecting areas with loss of volume, remodeling the contour of the bone bases, and correcting the wrinkles resulting from the aging process.^9^ Considering the efficiency and safety of the facial filling method with hyaluronic acid for aesthetic purposes in individuals without orofacial clefts, and the lack of contraindications related to the presence of clefts, the proposal of the present study was to evaluate the facial pleasantness in individuals with unilateral complete cleft palate who underwent facial fillers in the middle face with fillers based on hyaluronic acid (HA). Thus, this study aimed to evaluate the impact of orofacial harmonization with cross-linked hyaluronic acid on the facial pleasantness of patients with complete and unilateral cleft lip and palate, with skeletal Class III and mild to moderate maxillary deficiencies, rehabilitated with compensatory orthodontic treatment, without orthognathic surgery; and compare the assessment of evaluators with cleft lip and palate and orthodontists, as well as the anatomical structures identified as responsible for this judgment.

## MATERIAL AND METHODS

This prospective case control study was submitted for consideration by the research ethics committee of the Hospital for Rehabilitation of Craniofacial Anomalies (University of São Paulo, HRAC/USP, Department of Orthodontics, Bauru/SP, Brazil) and approved (n^o^ 4625926). The sample calculation of patients and groups of evaluators was based on the study by Rocha et al.^10^ Adopting a significance level of 5% and a power of 80%, to detect an effect size equal to 0.7 between evaluation times, on the pleasantness scale, a sample of 18 patients was required. The sample calculation for the necessary number of evaluators, considering a mean standard deviation between orthodontists with experience in clefts and lay people with clefts equal to 0.57 to determine the average pleasantness per group of evaluators, with a confidence level of 95% and a maximum error of 0.3, determined a minimum sample of 17 participants in each group.

The study group consisted of 18 individuals with complete and unilateral cleft lip and palate, permanent dentition, aged between 18 and 40 years (mean age 26.5 years), without signs of facial senility, of both sexes, with skeletal Class III and mild to moderate maxillary deficiency, absence of anterior crossbite and edge-to-edge bite, who have already completed interdisciplinary cleft rehabilitation, including compensatory orthodontic treatment without orthognathic surgery. All individuals in the sample were treated at the Hospital for Rehabilitation of Craniofacial Anomalies of the University of São Paulo, or in a private practice (Paranaguá, Brazil).

Exclusion criteria were: patients with chronic diseases, syndromes, pregnant women, obese, infants, allergic, keloid tendency, coagulation disorders, herpes, users of prescription drugs and infections in facial tissues, patients with beards, face piercing, tattoos, signs of aging on the face, freckles, active acne or atrophic acne scars. The evaluation of facial pleasantness was carried out by 36 examiners, divided into two groups: 18 orthodontists with at least two years of experience in orthodontic treatment of patients with orofacial clefts (14 women and 4 men) constituted the technical group called “Orthodontists Group” (OG); and 18 individuals with cleft lip and palate who did not receive the filler treatment (14 women and 4 men) constituted the group named “People with Clefts Group” (CLPG).

**Figure 1:**
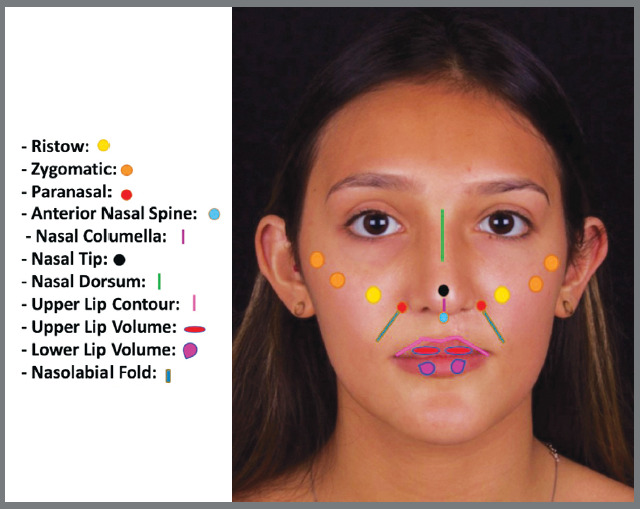
Front view of anatomical points filled with hyaluronic acid.

**Figure 2:**
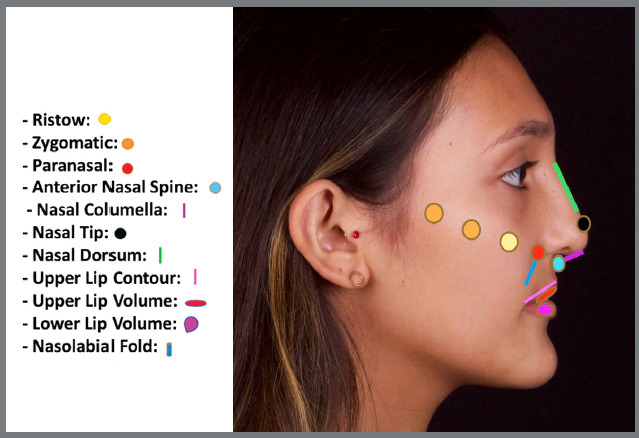
Profile view of anatomical points filled with hyaluronic acid.

After signing a free and informed consent form, the patients underwent a careful anamnesis and physical examination, to establish a protocol for facial harmonization procedures. Selected individuals underwent cross-linked hyaluronic acid facial fillers inserted exclusively in the midface, by a single operator. The lower lip was filled in when the patient had a flattened labial sulcus. The anatomical points filled in were: Ristow’s space, paranasal, anterior nasal spine, nasal dorsum, nasal tip, zygomatic arch, nasal columella, upper and lower lips, and and nasolabial fold (Figs 1 and 2).

All patients were anesthetized with an infraorbital nerve block, using local injectable anesthetic based on lidocaine hydrochloride 2% and felinephrine 1:100,000. Patients who underwent filling of the lower lip, in addition to filling of the middle third, were anesthetized with a mental nerve block. The dermal planes accessed in the fillings were supra periosteal in the Ristow space, paranasal and anterior nasal spine regions; and deep dermal in the other regions. In supra-periosteal regions, facial fillers based on hyaluronic acid with greater density (Rennova Ultra Deep^®^, Innovapharma Brasil Farmacêutica Ltda, Goiânia/GO, Brazil) were used, dispensed with #22G 50-mm cannula using the bolus technique.^11^ In the zygomatic arch, nasal columella, upper lip, nasal tip, and intermediate-density fillers (Rennova Lift^?^, Innovapharma Brasil Farmacêutica Ltda, Goiânia/GO, Brazil) were used. In the zygomatic arch, nasal columella, upper and lower lip, the HA was dispensed with #22G 50-mm cannula using the linear retroinjection technique.^11^ To fill the nasal tip, a #22G 50-mm cannula was used and the AH was dispensed using the bolus injection technique.^11^ The amounts of cross-linked hyaluronic acid used (ml) in each patient were defined after careful facial analysis, considering individual needs. The average volume of hyaluronic acid used per patient was 3.07 ml (Table 1).

**Table 1:** Quantity (ml) of material used in each structure.

Anatomical structure	Mean	S.D.
1) Ristow L	0.47	0.08
2) Paranasal R	0.41	0.12
3) Zygomatic R	0.17	0.21
4) Ristow L	0.47	0.12
5) Paranasal L	0.39	0.13
6) Zygomatic L	0.11	0.20
7) Nasolabial Sulcus R	0.03	0.14
8) Nasolabial Sulcus L	0.03	0.14
9) Anterior Nasal Spine	0.19	0.10
10) Nasal Dorsum	0.03	0.08
11) Nasal Tip	0.07	0.11
12) Nasal Columella	0.07	0.09
13) Upper Lip Contour	0.22	0.15
14) Upper Lip Volume	0.34	0.30
15) Lower Lip Volume	0.08	0.16
TOTAL	3.07	0.65

**Figure 3:**
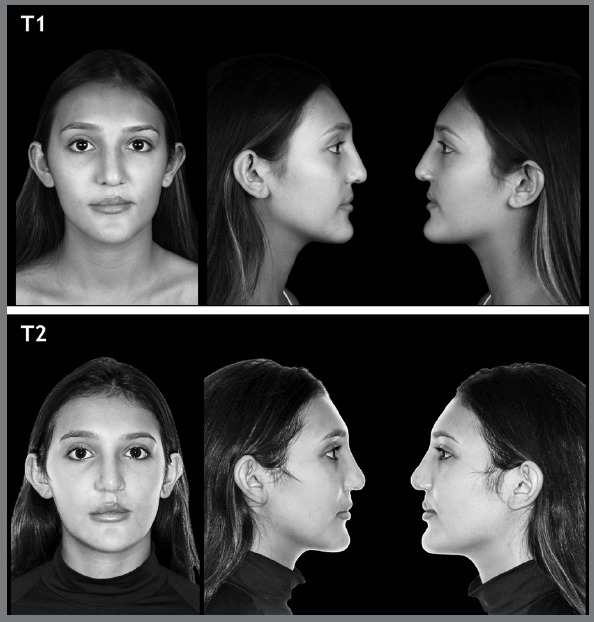
Pre and post-operative example of an individual undergoing facial filler in the middle face.

Three standardized photographs were taken (frontal norm at rest, right and left profile at rest) of each patient at two operative times (Fig. 3): T1) pre-filler, T2) 30 to 45 days post-filler. At the end of the study, each patient took a total of six photographs. Photographs were taken with the patients arranged in a natural head position, Frankfurt plane parallel to the floor, standing and looking at their own eyes reflected in a mirror 1.5 m away, without makeup, without accessories on the ears and neck, with lips at rest. The photographs of the patients in frontal norms at rest, right profile at rest, and left profile at rest at each time were grouped in this order and distributed on a page of a virtual album made on the Google Forms^®^ platform. Below each photograph, a Likert scale was placed for facial pleasantness assessment (Fig 2). In the same session of the virtual album, a list of eight anatomical structures was arranged so that the evaluator could mark which structure was responsible for choosing the option marked on the Likert scale. The “Other” field, placed below the list of anatomical structures, was enabled for the evaluators to fill in if they did not find the determining anatomical structure for their judgment.

The evaluators were instructed to disregard the ears and hair during the facial evaluation, so that the judgment of facial pleasantness was carried out considering the impacts on facial aesthetics provided only by facial fillers. To judge the facial pleasantness of each photograph, a Likert^12^ scale was used, in which: 1 is very unpleasant, 2 is unpleasant, 3 is acceptable, 4 is pleasant and 5 is very pleasant. Sheets with scales were made available under each photograph on every page of the album. The evaluators were instructed to mark an “X” in the option referring to their opinion on the scales arranged below each photograph. The evaluations were carried out at the two time-points: T1 and T2.

In addition to the evaluation of facial pleasantness performed using the Likert scale, the evaluators were asked which structure of the face was decisive for their judgment. The following options were arranged in the same section of the album, for the evaluators to choose from: cheekbone, nose, chin, upper lip, lower lip, dark circles, facial harmony, and profile. If the evaluators did not agree with the available response options, they could describe the determining structure for his judgment in the “other” tab.

### METHOD ERROR

To evaluate the intra-examiner agreement, 30% of the photographs in the frontal norms, right profile and left profile at T1 and T2 were randomly selected for a new evaluation. To measure the evaluation error, kappa statistics were used; whereas for the ordinal scale of pleasantness, linear weighting was used; and for the other structures, Kappa was used without weighting. The interpretation of the result was based on Landis and Koch.^13^

### STATISTICAL ANALYSES

Data were described by median, first quartile (Q1), third quartile (Q3), mean, and standard deviation (SD). The score of the pleasantness assessment scale of each patient was calculated by the median of the scores attributed by the evaluators. To verify the normality of data distribution, the Shapiro-Wilk test was used. As the distribution did not show normality, non-parametric tests were used in the analysis. For comparison between time-points T1 and T2, and between lay persons with cleft and orthodontists, the Wilcoxon test was used. To verify the correlation between the amount of material used and the pleasantness scale, Spearman’s correlation coefficient was used. In all tests, a significance level of 5% was adopted. All statistical procedures were performed using the SPSS software version 28.

## RESULTS

In the evaluation of the pleasantness scale, the Kappa of CLPG was 0.54 (moderate), and in the OG, it was 0.67 (substantial). In the evaluation of the structures, the CLPG resulted in Kappa of 0.22 (reasonable) and the OG, of 0.53 (moderate). Lay people with cleft lip and palate rated the face as more pleasant after the midface filling procedure. In the orthodontist’s perception, on the other hand, facial pleasantness remained similar after the facial filling procedure (Table 2).

**Table 2:** Comparison between T1 and T2, and between groups of evaluators, regarding the assigned agreeableness score (Kappa).

Evaluators	T1				T2				p
	Median	Q1	Q3	Mean	Median	Q1	Q3	Mean	
CLPG	3.00	2.00	3.50	2.89	3.25	2.50	4.00	3.25	0.008*
OG	3.00	2.00	3.00	2.86	3.00	3.00	4.00	3.11	0.083
p	0.655				0.212				

* Statistically significant difference (p<0.05). CLPG = cleft lip and palate group. OG = orthodontists group. Q1 = first quartile, Q3 = third quartile.

The comparison between the judgment of laypersons with cleft lip and palate and orthodontists did not show a difference before and after the facial harmonization procedure. However, facial pleasantness increased more in the judgment of the layperson with cleft lip and palate (Table 3). When the faces were classified as Pleasant or Very Pleasant, both groups considered the harmony of the face as the structure responsible for the judgment at T1. At T2, the group of laypeople with cleft described the profile, and the group of orthodontists described the harmony of the face. When the faces were considered Unpleasant or Very Unpleasant, both groups described the nose as the structure responsible for the judgment, both at T1 and T2 (Table 4).

**Table 3:** Comparison between the two groups of evaluators regarding the variation in the pleasantness score from T1 to T2.

Evaluators	Variation (T2-T1)			
	Median	Q1	Q3	Mean
CLPG	0.00	0.00	1.00	0.28
OG	0.00	0.00	0.00	0.08
p	0.059			

CLPG = cleft lip and palate group. OG = orthodontists group. Q1 = first quartile, Q3 = third quartile.

**Table 4:** Percentages of structures cited, separated by degree on pleasantness scale.

Anatomical Structure	Pleasant or Very pleasant				Acceptable				Unpleasant or Very unpleasant			
	CLPG		OG		CLPG		OG		CLPG		OG	
	T1 (%)	T2 (%)	T1 (%)	T2 (%)	T1 (%)	T2 (%)	T1 (%)	T2 (%)	T1 (%)	T2 (%)	T1 (%)	T2 (%)
CHEEKBONE	1.1	2.8	1.1	1.9	6.9	12.9	6.7	1.5	7.7	7.3	6.9	1.2
NOSE	5.3	4.2	18.2	11.4	16.1	18.8	22.4	30.4	31.0	53.1	39.2	48.8
CHIN	0.0	0.7	1.1	1.9	5.7	2.4	2.2	0.0	3.5	3.1	0.0	0.0
UPPER LIP	5.3	3.5	5.7	8.6	17.2	16.5	23.1	21.5	12.7	7.3	10.8	10.7
LOWER LIP	0.0	0.0	0.0	0.0	2.3	0.0	0.7	0.7	1.4	0.0	0.0	0.0
DARK CIRCLES	1.1	1.4	0.0	0.0	0.0	1.2	0.7	0.7	5.6	4.2	0.0	1.2
FACE HARMONY	54.7	38.5	54.5	58.1	27.6	24.7	16.4	17.8	15.5	14.6	15.7	9.5
PROFILE	32.6	48.3	18.2	17.1	24.1	23.5	25.4	25.2	22.5	10.4	24.5	26.2
EYES +	0.0	0.7	1.1	1.0	0.0	0.0	1.5	1.5	0.0	0.0	1.0	1.2
MIDFACE +	0.0	0.0	0.0	0.0	0.0	0.0	0.0	0.0	0.0	0.0	1.0	0.0
NASOLABIAL ANGLE +	0.0	0.0	0.0	0.0	0.0	0.0	0.7	0.0	0.0	0.0	1.0	1.2
FACE HEIGHT +	0.0	0.0	0.0	0.0	0.0	0.0	0.0	0.7	0.0	0.0	0.0	0.0

+ other. CLPG = cleft lip and palate group. OG = orthodontists group.

## DISCUSSION

The self-perception of facial appearance after reconstructive surgeries in patients with complete unilateral cleft lip and palate demonstrates that the structures best evaluated by the patients were the harmony of the total face, anterior teeth, and the facial profile; and the negative considerations of these individuals were related to the nose and upper lip, in that order.^14^ There are significant differences between the projection of the midface of individuals without orofacial clefts, when compared to the midface of individuals with cleft lip and palate who had access to the repairing procedure protocol.^14^ This evidence also reveals that the nose and upper lip were wider, larger, and flatter in patients with complete unilateral cleft lip and palate, compared to patients without clefts.^15^

This clear trend towards midface retrusion guided the selection of anatomical areas to be filled. When performing the technique, the primary objective was the projection of midface facial structures that are usually retroprojected in patients with unilateral complete cleft lip and palate. The detailed anatomical descriptions of the fat compartments in previous studies contributed to the understanding and selection of the anatomical points and planes that were filled in the present patients.^16^ These studies created a topographic map that guided the authors in the location and depth at which hyaluronic acid should be implemented, so that it would have an optimized performance, considering aesthetic gain and volume of the implanted product.^17^ In addition, the loss of projection of the midface through the aging process results from the retroposition of these points, and therefore they must be revolumized to adjust this projection of the midface.^16^ ^,^ ^17^

The facial expressions and anteroposterior maxillary projection can be influenced by filling in the Ristow points,^18^ which also contributes to the volumization of the lacrimal duct. This restored contour allows for a more anteriorly projected midface perception. The anterior projection of the paranasal region is directly influenced by maxillary deficiency and lack of projection of the anterior nasal spine in patients with unilateral complete cleft lip and palate. Unilateral cicatricial fibrosis resulting from reconstructive surgeries of the lip and nose still promotes an asymmetry of the anterior projection of this region.^19^ This trend means that, in some cases, fillers are applied in different amounts. The projection of Ristow’s point generally enhances the sensation of retropositioning of the paranasal region. With that, the volumization of this point becomes mandatory. From the authors’ perspective, the anterior projection of the midface would be finished by filling the Anterior Nasal Spine (ANS). The rebalancing of Ristow (R) point and the right and left paranasal (PN) points, located more superiorly in the midface, awakens the demand for support at its lowest point. Filling the anterior nasal spine completes the anterior projection of the midface and, when performed together with filling the nasal columella (NC), contributes to opening the nasolabial angle, which is often closed in individuals with clefts treated without orthognathic surgery. 

The points located on the zygomatic process of the maxilla were not filled in for all individuals in the sample. There is evidence that the faces of individuals with complete unilateral cleft lip and palate are wider when compared to the faces of individuals without cleft.^15^ Clinically we also had this feeling. Individuals who received facial fillers in the zygomatic area tended to have longer faces (dolichofacials) and therefore deserved an increase in transverse face perception. The filling of the nasolabial fold was also not performed in most individuals, as the support of the skin after filling the PN and R points softened the expression of this fold. Individuals who, after filling in the PN and R region showed the need to improve the aesthetic expression of the nasolabial fold, were submitted to filling in this region.

After the end of growth, individuals with complete unilateral cleft lip and palate tend to present the lower lip in front of the upper lip, due to maxillary deficiency and the lower thickness of the upper lip, a restriction imposed by cicatricial fibrosis.^20^ This tendency is also evidenced in skeletal Class III individuals without orofacial clefts and prognathic.^21^ The relationship between the lower lip and the front of the upper lip compromises facial aesthetics and therefore justifies its anterior projection by both filling in its contour and volume increase.

The filling of the lower lip was directed only to individuals who presented the flattening of the labial sulcus. In the present study, only three patients underwent this procedure, all female. Skeletal Class III individuals tend to have an open mentolabial angle, probably due to lower dental compensation.^21^ ^,^ ^22^ As the individuals selected in this study were treated without orthognathic surgery, the orthodontic compensatory approach was adopted, and this establishes a relationship between protruded upper incisors and retroclined lower incisors.^23^ This relationship is negative in the interpretation of facial aesthetics, especially in women.^24^ Slight volumization of the lower lip in Pattern III individuals restores the depth of the labial sulcus and harmonizes the contours of the lower third of the face. It should be pointed out that this approach was performed only in individuals who presented flattening of the labial sulcus, since the main purpose of the study was the management of the midface. In these cases, the volume of hyaluronic acid used in the upper lip was greater, as its anterior relationship to the lower lip should be achieved.

The most negative evaluations in the self-perception of facial aesthetics in rehabilitated patients with cleft were related to the nose.^14^ Fillers based on hyaluronic acid in this region were performed with the sole aim of refining the result of the surgical procedure. Using this technique in anticipation of major alterations is a mistake, but it can be useful for light retouching.^25^ In this sense, we used filling in the nasal dorsum to correct irregularities in the nasal hump, and in the nasal tip to lift the tip of the nose.

The rehabilitation of patients with cleft lip and palate aims to restore functional and aesthetic balance to individuals. The assessment of the functional benefits arising from rehabilitation, such as improvement in speech, improvement in the psychological aspect, and improvement in occlusion, is objective and can be measured by several methods already described.^26^ However, the evaluation of aesthetic benefits is subjective and can be influenced by gender, ethnicity, socioeconomic and cultural conditions, and quality of evaluators (technicians, laypeople), among other variables.^12^ ^,^ ^26^ Studies that involve subjective measures have some inner difficulties related to the variables to be judged with ideal responses, which are not always objective. This limitation is compensated with the use of scales, as they allow the evaluator to grade his response considering two extremes. The use of scales is employed in studies on pain, and quality of life, among other occurrences of a subjective nature.^27^ The use of the Likert scale and the visual analog scale (VAS) has been applied in studies that evaluate facial pleasantness in patients with cleft lip and palate.^26^ A comparative study between the Likert and VAS scales, when used to assess facial pleasantness, concluded that both methods are effective and can be used for this purpose; however, the evaluators showed a preference for the LIKERT scale, due to the simplicity of recording responses and representing subjective opinions.^12^ The Likert scale proved to be easy for the evaluators to understand and efficient, when considering the time used for the evaluation. In addition, the practicality of preparing the statistics made us conclude that this method is reproducible.

The group of evaluators was formed by individuals with academic training and experience in the area of clefts, and lay people with cleft, similarly to previous studies.^26^ The results obtained in the evaluation of the facial aesthetics of individuals with cleft lip and palate, carried out by lay individuals with clefts, brought researchers closer to the understanding of experiences, perspectives, self-perception, satisfaction, disappointments with the treatment, and may contribute to the development of new strategies.

The self-perception of facial aesthetics of individuals with cleft is the most positive, when compared to evaluators with experience in cleft, parents and lay people without cleft.^28^ This predisposition was also observed in our results. Evaluators with cleft noticed changes between T1 and T2, and scored increased facial pleasantness. This may be the result of a psychological mechanism that tends to increase an individual’s self-confidence and resilience and support their social position.^28^

Orthodontist evaluators with cleft experience were not sensitized to the point of increasing facial pleasantness scores between T1 and T2. Some studies indicate that these evaluators are more flexible when considering facial pleasantness in patients with CLP.^30^ The understanding of treatment limitations may explain this compliance and justify those more positive evaluations. However, in our research, the orthodontist evaluators did not judge the faces with less rigor. We can also speculate that some individuals who received compensatory orthodontic treatment, due to their anatomical possibilities, should have been indicated for treatment with orthognathic surgery to improve facial relationships. Thus, we assume that facial fillers have become irrelevant in the eyes of orthodontist evaluators, given the magnitude of the dental-skeletal imbalance of some patients.

The faces considered unpleasant or very unpleasant by both groups, both in T1 and T2, had the nose as the structure responsible for the negative judgment. This means that the disharmony of the nose impressed the evaluators to the point of not being sensitized by other anatomical structures of the midface that approached balance after facial filling, such as the profile and facial harmony. 

When they classified the faces as Pleasant or Very Pleasant, the most valued structure in T1, by both groups, was the harmony of the face. The interpretation of this data is that when the face does not present the imbalance of an isolated anatomical unit, the pleasantness comes from the evaluation of the set of anatomical structures. At T2, the group of laypeople with a cleft was sensitized by the profile adjustment, but the group of orthodontists continued to point out the harmony of the face as responsible for the positive judgment. This inertia in the judgment of the technical group was interpreted as a lack of awareness of these evaluators regarding the impact of facial fillers.

When nose rehabilitation is not favourable, hyaluronic acid-based facial fillers are not enough to remove the individual’s face from a negative judgment and make it acceptable or pleasant. During sample collection, we observed that not all patients had undergone surgical rhinoplasty. Although these individuals were under our selection criteria, as they had already completed the rehabilitation process, they remained unsatisfied and looking for a new surgical procedure to correct slight imperfections. We consider this fact to be a limitation of the study, as we know the value given to the relationship between nose aesthetics in the judgment of facial pleasantness by our group of evaluators. That said, we suggest that new studies be developed with rehabilitated individuals and with good nose aesthetic relationships. This would make the evaluators’ judgment more directed towards the effectiveness of the facial filling in restoring facial aesthetics.

## CONCLUSIONS

» Filling the middle third of the face in patients with cleft lip and palate treated without orthognathic surgery increased the pleasantness of the face in the opinion of laypeople with cleft lip and palate.
» Orthodontists with experience in cleft lip and palate care were not sensitive to the facial alterations caused by the facial filling procedure.
